# Reflection on modern methods: statistical, policy and ethical implications of using age-standardized health indicators to quantify inequities

**DOI:** 10.1093/ije/dyab132

**Published:** 2021-07-05

**Authors:** Katherine A Thurber, Joanne Thandrayen, Raglan Maddox, Eden M Barrett, Jennie Walker, Naomi Priest, Rosemary J Korda, Emily Banks, David R Williams, Raymond Lovett

**Affiliations:** 1 National Centre for Epidemiology and Population Health, Australian National University, Acton ACT, Australia; 2 Centre for Social Research and Methods, College of Arts and Social Sciences, Australian National University, Acton ACT, Australia; 3 Population Health, Murdoch Children’s Research Institute, Royal Children’s Hospital, Parkville, VIC, Australia; 4 Sax Institute, Ultimo, NSW, Australia; 5 Department of Social and Behavioral Sciences, Harvard T. H. Chan School of Public Health, Boston, MA, USA; 6 Department of African and African American Studies, Harvard University, Cambridge, MA, USA

**Keywords:** Reference standards, benchmarking, age distribution, racism, health inequity

## Abstract

Methods for calculating health indicators profoundly influence understanding of and action on population health and inequities. Age-standardization can be useful and is commonly applied to account for differences in age structures when comparing health indicators across groups. Age-standardized rates have well-acknowledged limitations, including that they are relative indices for comparison, and not accurate measures of actual rates where the age structures of groups diverge. This paper explores these limitations, and demonstrates alternative approaches through a case study quantifying mortality rates within the Aboriginal and Torres Strait Islander (Indigenous) population of Australia and inequities compared with the non-Indigenous population, over 2001–16. Applying the Australian Standard Population, the Aboriginal and Torres Strait Islander age-standardized mortality rate was more than double the crude mortality rate in 2001 and 2016, inflated through high weighting of older age groups. Despite divergent population age structures, age-standardized mortality rates remain a key policy metric for measuring progress in reducing Indigenous-non-Indigenous inequities in Australia. Focusing on outcomes age-standardized to the total population can obscure inequities, and denies Aboriginal and Torres Strait Islander peoples and communities valid, actionable information about their health and well-being. Age-specific statistics convey the true magnitude of health risks and highlight high-risk subgroups. When requiring standardization, standardizing to a population-specific standard (here, an Indigenous standard) generates metrics centred around and reflective of reality for the population of focus, supporting communities’ self-determination to identify priorities and informing resource allocation and service delivery. The principles outlined here apply across populations, including Indigenous and other populations internationally.

Key Messages

- Measurement of inequities should be designed to serve the needs of the population of interest.
- Age-standardization is commonly used when comparing health indicators across groups and populations, despite known limitations when the populations have divergent age structures.
- Standardizing outcomes to the age distribution of other populations can deny the population of focus accurate information about their health and well-being, and reinforce structural racism.
- In contrast, age-specific statistics convey the true magnitude of health risks and highlight high-risk subgroups in the population(s) of focus.
- When standardization is required, standardizing to a population-specific standard (such as an Indigenous standard) generates metrics centred around and reflective of reality for the population of focus, supporting self-determination and Indigenous data sovereignty principles.

## Introduction

Monitoring of trends in health and inequities is critical to informing local and national decision making.^1–4^ Accurate, relevant and appropriate measures are required for this to be effective.^2–4^ Measurement of inequities should be designed to serve the needs of the population of focus.

Age-standardized rates are commonly used globally to compare event rates among populations and groups with different age structures*.* Although providing a common metric for comparison, they are not accurate measures of actual event rates where populations have divergent age structures. There are well-established limitations to using age-standardized rates to inform policy, including that these average measures may not convey the true magnitude of risks or inequities (but are often misinterpreted as doing so), are relatively insensitive to change and provide limited insight into targets for programmes and policies.^5–8^ In addition, age-standardized rates can vary dramatically depending on which Standard Population is used, and standardizing outcomes to the age distribution of another population denies the population of focus accurate information about their health and well-being. Despite these major limitations, age-standardized rates remain commonly calculated and reported across populations without adequate context, suggesting that the implications of their limitations are under-appreciated.

The purpose of this article is to demonstrate the statistical, policy and ethical implications of using age-standardized metrics to quantify inequities between populations with divergent age structures, through a case study of Aboriginal and Torres Strait Islander and non-Indigenous mortality rates from Australia. This article presents approaches for quantifying mortality and mortality inequities—age-specific rates and use of a population-specific standard—which more accurately reflect the experiences and needs of the population of focus, thereby generating valid evidence for decision making and planning.

Ethics approval was not required because this secondary analysis was based on aggregated publicly available data. The work was conducted in line with principles for the ethical conduct of Aboriginal and Torres Strait Islander health research.

## Context

In Australia, Aboriginal and Torres Strait Islander (Indigenous) peoples have experienced health and social inequities compared with the total population since colonization.^9^^,^^10^ In response to advocacy from Aboriginal and Torres Strait Islander peoples, communities and organizations, in 2008, the Council of Australian Governments committed to *Closing the Gap in Indigenous Disadvantage* compared with non-Indigenous Australians.^11^^,^^12^

The primary target under the *Closing the Gap* strategy is to close the life expectancy gap within a generation.^11^ Under the associated National Indigenous Reform Agreement (NIRA), average life expectancy at birth is the primary performance indicator for this target.^13^ Life expectancy estimates are only available 1 out of every 5 years; when these are unavailable, progress against this target is based on age-standardized mortality rates (ASMRs).^13^ Therefore, age-standardized measures are the focus of most annual government reporting against this target.

## Measuring mortality inequities

The crude mortality rate in a population (i.e. total number of deaths per 100 000 population per year) reflects the average risk of mortality, across all ages, in a population. An age-specific mortality rate is the mortality rate observed within a specific age group. Because mortality rates are strongly tied to age (e.g. death rates tend to be highest in the oldest age group), crude mortality rates in two populations are not immediately comparable where there are differences in the age composition of the populations.

Direct age-standardization removes age composition differences by applying the age-specific mortality rate observed in the population(s) of interest to the age distribution of a reference population, called the Standard Population. This creates a summary measure of mortality, the age-standardized mortality rate (ASMR), which is a weighted sum of age-specific mortality rates. ASMRs based on the same Standard Population can be compared across populations and over time,^8^^,^^14^^,^^15^ using rate ratios (RRs; a relative measure) and rate differences (RDs; an absolute measure).

### Established statistical limitations of age-standardized rates

ASMRs do not reflect the true mortality rates for a population; they are hypothetical values, informative only as a means of comparison.^14–19^ However, they are commonly misinterpreted as actual mortality rates by policy makers, researchers and the public.^5^^,^^15^^,^^19^ Comparison of ASMRs using RRs and RDs can further mask the ‘fictions’ underpinning these statistics.^19^

The choice of a Standard Population can affect the sizes of ASMRs, RRs and RDs and therefore can materially alter interpretation of comparisons.^1^^,^^4^^,^^8^^,^^14^^,^^15^^,^^17^^,^^19–21^ For example in the USA, changing from the 1940 Standard Population to the 2000 Standard Population resulted in apparent decreases in racial inequities according to age-standardized mortality RRs. Using the 1940 Standard Population, the age-standardized mortality RR for Black Americans compared with White Americans was 1.6 in 1995, unchanged from 1.6 in 1950. Applying the 2000 Standard Population resulted in an age-standardized RR of 1.4 for 1995; this apparent decline in RR was an artefact of the older age composition of the 2000 versus the 1940 Standard Population.^14^^,^^22^ The incorrect interpretation of the observed change— based on use of a different Standard Population—as a sign of decreasing inequities could have substantial implications, including deprioritization of programmes and policies for these groups, which could in turn exacerbate inequities.^4^^,^^17^

A key statistical consideration in selecting a standard that can generate data meaningful to the population of interest is that the age distribution be relatively aligned with that of the population of interest.^14^^,^^17–19^^,^^23^^,^^24^ Selecting a standard that is meaningful to the population of interest (here, Aboriginal and Torres Strait Islander peoples) is consistent with the Indigenous Data Governance protocols and principles, and the United Nations Declaration on the Rights of Indigenous Peoples (Article 31), supporting self-determination.

Acknowledging the aforementioned limitations, specific principles were developed in Australia for age-standardization in measuring Indigenous: non-Indigenous inequities,^25^ and in *Closing the Gap* policy reporting, the NIRA specifies that these principles are to be followed.^14^ The overarching principle is that ‘Before undertaking age-standardization, analysts must investigate the data being used to understand the age-specific distribution and any limitations that may impact on the results’.^25^ Consistent with statistical advice,^14^^,^^18^ the principles require the additional reporting of age-specific measures when ‘the age-standardized rates and rate ratios lie largely outside the range of the age-specific rates and rate ratios’ (Principle 5a).^25^

Regardless of the Standard Population used, standardized statistics are a summary measure, and ‘may actually conceal more than they reveal’.^17^ Aggregating all age groups into a single summary statistic is likely to combine groups with disparate mortality rates, creating an imprecise estimate that obscures differences.^14^^,^^15^ Aggregated statistics therefore are not sensitive to change.

Age-specific mortality rates reflect the actual experience of the population: they reflect the true magnitude of health risks and highlight high-risk subgroups—information with clear policy implications and value to communities. Compared with ASMRs, age-specific rates are more sensitive to change, enabling more timely detection of progress where it has occurred.

### Ethical considerations regarding use of age-standardized mortality rates

Standardizing Aboriginal and Torres Strait Islander outcomes to the age distribution of the total Australian population privileges the experience of the non-Indigenous majority (97%). Aboriginal and Torres Strait Islander peoples have the right to real, accurate and clearly articulated information about their health and well-being,^3^^,^^26^^,^^27^ but are denied this through the focus on ASMR in reporting. Further, the population chosen as the Standard Population may be seen to ‘signify which population is considered “normal” or central’.^4^ p. 339 In Australia, the Standard Population is defined as the national population at 30 June 2001,^25^ which principally reflects the non-Indigenous population. This frames the Aboriginal and Torres Strait Islander population as ‘other’, contributing to deficit discourse^1^^,^^2^^,^^28^ and reinforcing structural racism.

Researchers in Aotearoa (New Zealand) have proposed and have used an Indigenous Standard for quantifying Māori-non-Māori inequities.^1^^,^^3^^,^^4^^,^^29^ This strengths-based approach centres the analysis on the Indigenous population, and generates standardized mortality rates that approximate the crude (actual) mortality rates experienced by that population. The use of an Indigenous Standard is consistent with calls from Indigenous populations in other countries^1^^,^^3^ and with human rights^26^^,^^27^ and statistical and data- reporting principles.^13^^,^^14^^,^^18^^,^^25^ To our knowledge, an Indigenous Standard has not been used to age-standardize metrics for the Aboriginal and Torres Strait Islander population.

## Worked example

We examine the age structure of the Aboriginal and Torres Strait Islander and non-Indigenous populations, compared with the Australian Standard and an Indigenous Standard (here, the national 2016 Aboriginal and Torres Strait Islander Estimated Resident Population).

Population estimates and deaths (all-cause) by age group for the Aboriginal and Torres Strait Islander population and the non-Indigenous population were extracted from Australian Bureau of Statistics information (ABS Stat). Data are restricted to the five of eight Australian States/Territories where the quality of Indigenous identification is deemed acceptable. Data were extracted for 2001, 2006, 2011 and 2016, to align with national Census years; data preceding 2001 were not available. We calculated crude and age-specific mortality rates. We then calculated ASMRs using the Australian Standard and an Indigenous Standard. We examined absolute and relative changes in age-specific mortality rates and in ASMRs over 2001–16, within the Aboriginal and Torres Strait Islander population and between the Aboriginal and Torres Strait Islander and non-Indigenous populations. All mortality rates reported are per 100 000 population. Detailed data and methods are presented in Supplementary Material, available as Supplementary data at *IJE* online.

### Age distribution

**Figure 1:**
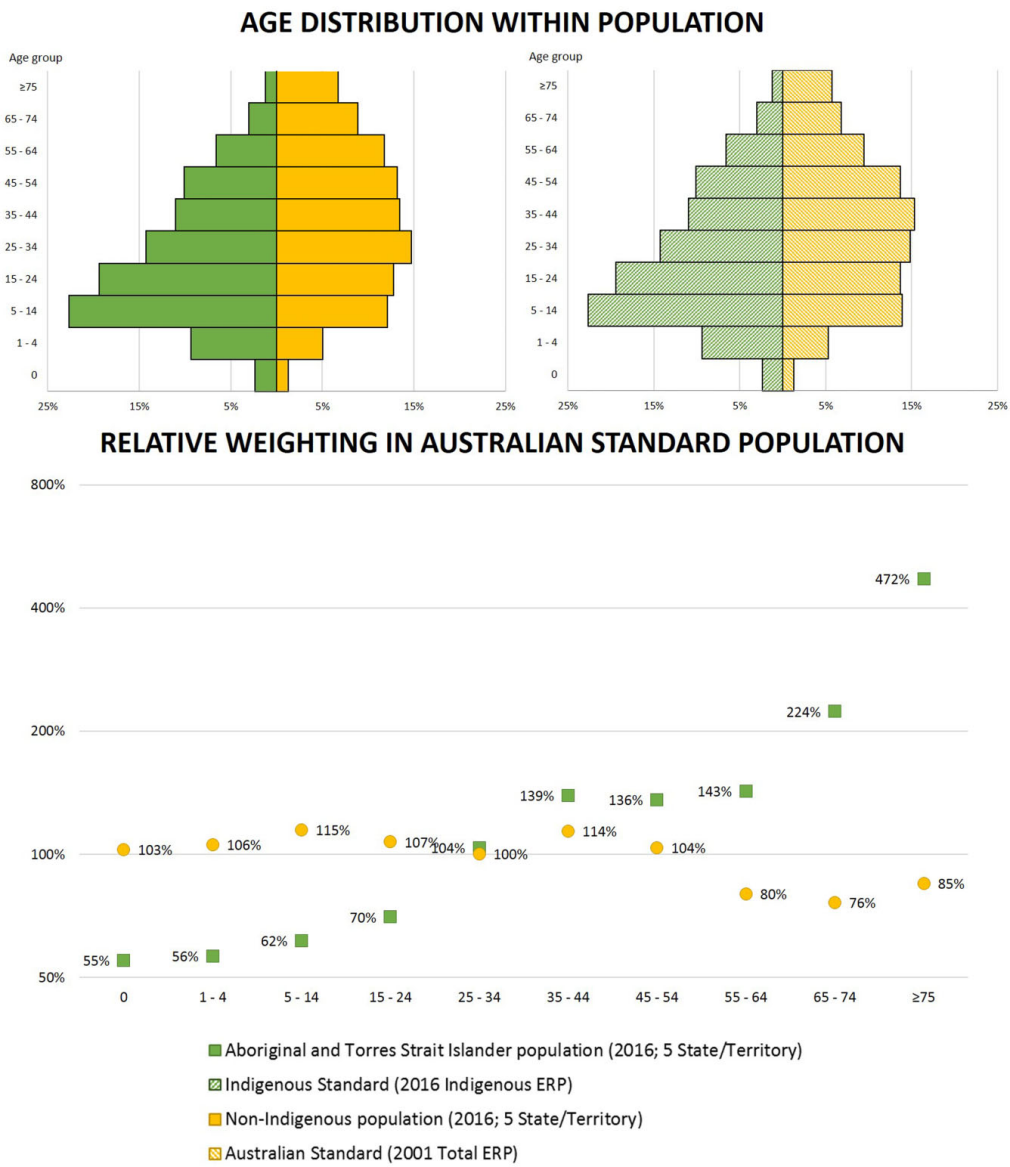
Age distribution of the 2016 Aboriginal and Torres Strait Islander and non-Indigenous population, the Australian Standard, and an Indigenous Standard, and relative weighting in standardization to the Australian Standard Population. Data are restricted to the five of eight Australian States/Territories where the quality of Indigenous identification is deemed adequate (New South Wales, Queensland, South Australia, Western Australia, Northern Territory).

The 2016 five-State/Territory Aboriginal and Torres Strait Islander population age distribution mirrors that of the Indigenous Standard (Figure 1); they are not exactly the same because the population used for calculating mortality is restricted to the five of eight States/Territories where the quality of identification is deemed acceptable, whereas the Indigenous Standard includes the total population nationally from all eight States/Territories (see Supplementary Material). The age distribution is markedly younger than the Australian Standard Population and therefore, when standardized to the Australian Standard Population, Aboriginal and Torres Strait Islander deaths in younger age groups are under-represented (low weighting applied) and Aboriginal and Torres Strait Islander deaths in older age groups are over-represented (high weighting applied), compared with their actual occurrence. This divergence of age structure calls into question the validity of using the Australian Standard for analyses designed to provide insights about the Aboriginal and Torres Strait Islander population.

### Crude mortality rates

We observed no material change in the crude mortality rate for the Aboriginal and Torres Strait Islander population from 2001 to 2016 (409 deaths per 100 000 in 2001 to 414 in 2016) (Table 1; Supplementary Table S3, available as Supplementary data at *IJE* online). The crude mortality rate for the non-Indigenous population was 655 in 2001 and 665 in 2016.

**Table 1:** Aboriginal and Torres Strait Islander and non-Indigenous age-specific and age-standardized mortality rates in 2001 and 2016, within-population rate differences and rate ratios for 2016 versus 2001, and Indigenous-non-Indigenous rate differences and rate ratios for 2001 and 2016, by age group

Persons	Aboriginal and Torres Strait Islander mortality rate (95% CI), and change 2001 to 2016				Non-Indigenous mortality rate (95% CI), and change 2001 to 2016				Indigenous versus non-Indigenous RD		Indigenous versus non-Indigenous RR	
	2001	2016	RD	RR	2001	2016	RD	RR	2001	2016	2001	2016
Age-specific mortality rates												
0	823 (680, 987)	591 (480, 721)	−232 (−420, −44)	0.72 (0.54, 0.95)	492 (459, 527)	286 (263, 310)	−206 (−245, −166)	0.58 (0.52, 0.65)	331 (208, 455)	305 (218, 393)	1.67 (1.37, 2.04)	2.07 (1.65, 2.56)
1–4	49 (32, 71)	23 (13, 37)	−26 (−47, −5)	0.46 (0.23, 0.90)	25 (21, 29)	16 (13, 19)	−9 (−14, −4)	0.64 (0.50, 0.81)	24 (10, 38)	7 (−3, 17)	1.96 (1.26, 2.96)	1.43 (0.78, 2.44)
5–14	17 (10, 26)	19 (13, 27)	2 (−8, 12)	1.12 (0.61, 2.08)	13 (11, 14)	8 (7, 9)	−5 (−7, −3)	0.61 (0.49, 0.75)	4 (−3, 10)	11 (6, 16)	1.33 (0.80, 2.10)	2.44 (1.59, 3.63)
15–24	122 (100, 149)	77 (63, 93)	−46 (−72, −19)	0.63 (0.47, 0.83)	55 (52, 59)	34 (31, 36)	−22 (−26, −17)	0.61 (0.55, 0.67)	67 (51, 84)	43 (33, 54)	2.22 (1.79, 2.73)	2.29 (1.85, 2.82)
25–34	231 (198, 267)	197 (170, 226)	−34 (−77, 10)	0.85 (0.69, 1.05)	72 (68, 76)	52 (49, 55)	−20 (−24, −15)	0.73 (0.67, 0.79)	159 (139, 179)	145 (130, 160)	3.22 (2.73, 3.77)	3.77 (3.23, 4.39)
35–44	456 (404, 514)	358 (317, 402)	−98 (−166, −31)	0.78 (0.66, 0.93)	113 (109, 118)	98 (94, 102)	−15 (−21, −9)	0.87 (0.82, 0.92)	343 (314, 372)	260 (236, 283)	4.03 (3.54, 4.57)	3.65 (3.21, 4.13)
45–54	784 (696, 880)	644 (586, 706)	−140 (−245, −35)	0.82 (0.71, 0.96)	235 (228, 242)	210 (204, 216)	−25 (−34, −16)	0.89 (0.86, 0.93)	549 (497, 600)	434 (398, 469)	3.33 (2.95, 3.75)	3.07 (2.78, 3.38)
55–64	1863 (1669, 2073)	1262 (1162, 1369)	−600 (−806, −394)	0.68 (0.59, 0.78)	620 (606, 634)	486 (477, 496)	−134 (−150, −117)	0.78 (0.76, 0.81)	1243 (1125, 1360)	776 (711, 842)	3.00 (2.69, 3.35)	2.60 (2.38, 2.82)
65–74	3355 (2998, 3743)	2536 (2326, 2759)	−819 (−1223, −416)	0.76 (0.66, 0.87)	1744 (1717, 1771)	1178 (1160, 1196)	−566 (−597, −535)	0.68 (0.66, 0.69)	1611 (1344, 1878)	1358 (1209, 1506)	1.92 (1.72, 2.15)	2.15 (1.97, 2.35)
75 and over	7514 (6673, 8432)	7356 (6786, 7961)	−158 (−1198, 881)	0.98 (0.85, 1.13)	6778 (6721, 6837)	6576 (6529, 6624)	−202 (−277, −127)	0.97 (0.96, 0.98)	736 (−89, 1561)	780 (227, 1332)	1.11 (0.98, 1.24)	1.12 (1.03, 1.21)
Mortality rates for all ages combined												
Crude	409 (391, 428)	414 (399, 429)	5 (−19, 28)	1.01 (0.96, 1.07)	655 (651, 660)	665 (661, 669)	9 (3, 15)	1.01 (1.01, 1.02)	−246 (−269, −223)	−250 (−270, −231)	0.62 (0.60, 0.65)	0.62 (0.60, 0.65)
Age-standardized (Indigenous Standard)	530 (506, 555)	418 (403, 433)	−113 (−141, −84)	0.79 (0.73, 0.85)	251 (248, 253)	204 (202, 206)	−47 (−49, −44)	0.81 (0.80, 0.83)	280 (255, 304)	214 (199, 229)	2.12 (2.07, 2.16)	2.05 (2.01, 2.09)
Age-standardized (Australian Standard)	1079 (1019, 1142)	908 (869, 948)	−171 (−244, −98)	0.84 (0.78, 0.91)	644 (639, 648)	565 (562, 569)	−78 (−84, −73)	0.88 (0.87, 0.89)	436 (374, 497)	343 (303, 382)	1.68 (1.62, 1.73)	1.61 (1.56, 1.65)

All mortality rates are per 100 000 population. Mortality rate data are restricted to the five of eight Australian States/Territories where the quality of Indigenous identification is deemed adequate (New South Wales, Queensland, South Australia, Western Australia, Northern Territory).

95% CI, 95% confidence interval; RD, rate difference; RR, rate ratio.

### Age-specific mortality rates

Examination of age-specific mortality rates provides evidence of mortality rate reductions from 2001 to 2016 for Aboriginal and Torres Strait Islander people aged 15–24, 35–44, 55–64 and 65–74 years. These mortality rate reductions were substantial in some cases, such as an absolute decrease of 819 deaths per 100 000 people aged 65–74 years (from 3355 in 2001 to 2536 in 2016), and an almost 40% reduction in mortality rate for those aged 15–24 years (RR = 0.63, from 122 in 2001 to 77 in 2016).

When the Indigenous-non-Indigenous ‘gap’ in age-specific mortality rates is assessed in absolute terms (according to RD), findings are generally consistent with a narrowing ‘gap’ from 2001 to 2016 for most age groups. This reflects greater absolute declines in mortality rates within the Aboriginal and Torres Strait Islander compared with non-Indigenous population for these groups. In contrast, if the ‘gap’ is examined in relative terms (according to RR), there is not a clear pattern in the change across age groups.

### Age-standardized rates, RRs and RDs

When age-standardized to the Indigenous Standard, the ASMR for the Aboriginal and Torres Strait Islander population was 530 per 100 000 in 2001 and 418 in 2016, reflective of observed crude mortality rates. When age-standardized to the Australian Standard, the Aboriginal and Torres Strait Islander ASMR was 1,079 per 100 000 in 2001 and 908 in 2016. These ASMRs are more than double the crude mortality rate. The Aboriginal and Torres Strait Islander ASMR is inflated through the high weighting of the 65–74- and ≥75-year age groups when using the Australian Standard Population.

The choice of Standard Population does not alter the assessment that the Aboriginal and Torres Strait Islander mortality rate declined from 2001 to 2016. In absolute terms, the ASMR declined by 113 [95% confidence interval (CI): 84, 141] deaths per 100 000 when the Indigenous Standard was used, or by 171 (95% CI: 98, 244) when the Australian Standard was used. Similarly, in relative terms, use of the Indigenous Standard identifies a 21% (RR = 0.79; 95% CI: 0.73, 0.85) reduction in ASMR over the period, and the Australian Standard a 16% (RR = 0.84; 95% CI : 0.78, 0.91) reduction.

When focusing on the Indigenous-non-Indigenous ‘gap’, use of the Indigenous Standard versus Australian Standard identifies a smaller ‘gap’ in absolute terms at each time point (RD ranging from 214 to 280 across years, compared with 343 to 436). When the Australian Standard is used, the absolute ASMR ‘gap’ is close to the total crude mortality rate observed within the Aboriginal and Torres Strait Islander population. In contrast, use of the Indigenous Standard versus Australian Standard identifies a greater ‘gap’ in relative terms (RR = 2.05–2.12 versus RR = 1.61–1.68). Use of an Indigenous Standard (compared with the Australian Standard) increases the RR, as it gives proportionate weight to the young and middle-aged groups, who are under-represented in the Australian Standard Population and where age-specific Indigenous-non-Indigenous RRs are highest. Accordingly, it gives lesser weight to the oldest age groups, where absolute death rates and RDs are highest. This is consistent with findings from New Zealand, where use of the Māori Standard led to larger Māori-non-Māori RRs, but smaller RDs, compared with use of the WHO Standard.^1^

Regardless of the Standard Population used, findings are consistent with a decrease in the Indigenous-non-Indigenous ASMR ‘gap’ from 2001 to 2016 in absolute terms, and no material change in the ‘gap’ in relative terms (Table 1). Therefore, assessment of progress against the policy target is not altered by the change in Standard. Most government reporting focuses on Indigenous-non-Indigenous standardized mortality RRs, and according to this measure, use of the Australian (compared with Indigenous) Standard has underestimated the ‘gap’. If the magnitude of inequity is greater than previously understood, resourcing should be increased commensurately.

**Figure 2.**
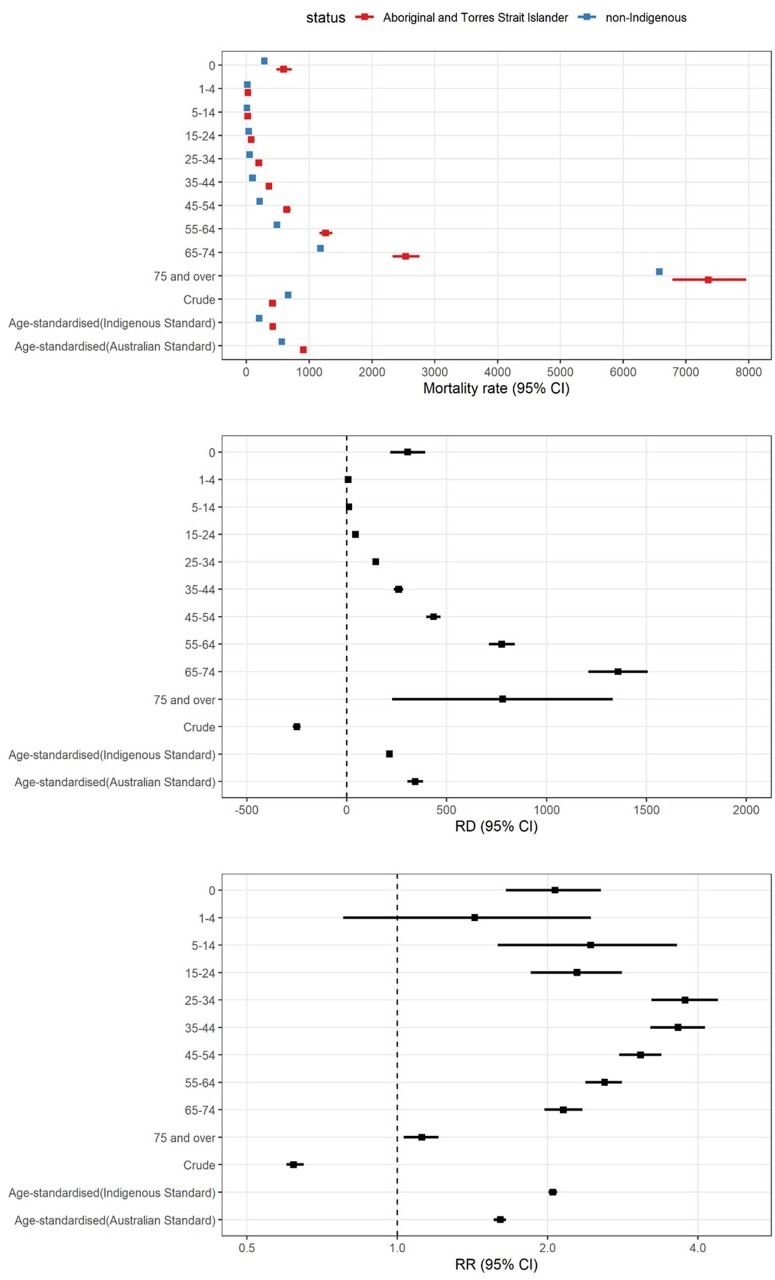
2016 age-specific, age-standardized, and crude mortality rates in the Aboriginal and Torres Strait Islander population and non-Indigenous population, and rate ratios (RRs). All mortality rates are per 100 000 population. Mortality rate data are restricted to the five of eight Australian States/Territories where the quality of Indigenous identification is deemed adequate (New South Wales, Queensland, South Australia, Western Australia, Northern Territory)

In all periods examined, the standardized metrics do not reflect age-specific mortality rates. This reflects a situation where, according to the established principles, age-specific measures must also be presented if the ASMR is reported.^25^ For example, the 2016 ASMR (908 using the Australian Standard) is not an informative summary of Aboriginal and Torres Strait Islander age-specific mortality rates, which range from 19 to 7356 (Figure 2). The 2016 Indigenous-non-Indigenous standardized mortality RR was 1.61(95% CI: 1.56, 1.65) when the Australian Standard was used, representing a 60% higher overall mortality rate in the Aboriginal and Torres Strait Islander population. This does not reflect the age-specific Indigenous-non-Indigenous RRs, which show that mortality is 12% higher for ≥75-year-olds and 43% higher for 1–4-year-olds, but more than double for all other age groups, up to nearly 4-fold for those aged 25–34 years. Using the Indigenous Standard generates an Indigenous-non-Indigenous age-standardized mortality RR (2.05 in 2016) that better fits within the range of age-specific RRs (1.12–2.77 in 2016), but still obscures variation within the population.

## Discussion

Large-scale data and statistics profoundly influence policy, with potential to alter life opportunities. Through this case study we demonstrate the importance of using statistics that are centred around and more accurately reflect the experience of the population of focus. This can be achieved by using age-specific rates or, where a summary statistic is required, by standardizing rates to the age distribution of the population of interest—rather than to that of another population.

The initial Australian *Closing the Gap* strategy and targets were defined for 2008–18, and the ongoing strategy has been refined.^30^ The primary target remains the same, but methods for assessing progress against the target have not been published at the time of writing.^30^ This presents an opportunity to revise the approach to focus on age-specific mortality rates and ASMRs standardized to an Indigenous Standard. Use of the Indigenous Standard would not materially change our assessment of progress in closing the ‘gap’, but would provide Aboriginal and Torres Strait Islander communities with real data about their population, supporting self-determined action.

If an Indigenous Standard were to be introduced in any country, use of the most current population estimates is recommended.^17^ However, there are complexities in introducing any new Standard Population,^1^ including that ASMRs calculated using different Standard Populations are not comparable.^14^ The potential implications of a change in Standard Population should be discussed with the population of focus and with peak statistical agencies, and any change should be communicated clearly to stakeholders.^14^

More broadly, consistent with principles of strengths-based reporting, it is important to question the benefit of comparing one population (e.g. an Indigenous population) with another (e.g. a non-Indigenous population), and to consider the potential harms of doing so.^2^^,^^28^ As an alternative to between-population comparisons, observed age-specific mortality rates within the population could be compared with evidence-based benchmarks representing best attainable health—rather than setting the health status of another population (e.g. a non-Indigenous population) as the goal to strive towards.

## Supplementary Data

Supplementary data are available at *IJE* online.

## Funding

This work was supported by the National Health and Medical Research Council of Australia [1156276 to K.T., 1122273 to R.L., 1136128 to E.B.].

## Data availability

Population and mortality data from the ABS are publicly available at [http://stat.data.abs.gov.au/Index.aspx].

## Conflict of Interest

None declared.

## Supplementary Material

dyab132_Supplementary_Data
