# Supplementary material for: Reflection on modern methods: statistical, policy and ethical implications of using age-standardized health indicators to quantify inequities
Source: Int J Epidemiol. 2021 Jul 5;51(1):324–33. doi: 10.1093/ije/dyab132 (PMC8855998; doi:10.1093/ije/dyab132)
Supplement: dyab132_Supplementary_Data [file dyab132_supplementary_data.docx]

SUPPLEMENTARY MATERIAL

Supplementary Material 1: Details on data sources and statistical analysis

Data for the Aboriginal and Torres Strait Islander and non-Indigenous populations were restricted to the five States and Territories (New South Wales, Queensland, South Australia, Western Australia, Northern Territory) where the quality of Indigenous identification in deaths data is deemed adequate. The ABS make minor adjustments to small cells to protect confidentiality; as a result, our calculations presented here based on the adjusted deaths figures may differ slightly from reported mortality rates based on non-adjusted figures.

Confidence intervals (CIs) for age-specific mortality rates were calculated using Poisson distribution,(**1**) and CIs for ASMRs were calculated using Gamma distribution.(**2**) CIs around RR and RD for age-specific rates were calculated using the Exact Poisson Method and Test Based Method respectively.(**3**) A Log-Normal and Normal CI were computed for RR and RD of ASMRs respectively.

Analysis was undertaken in R version 3.6.2 (package dsr(**4**)) and MedCalc.(**5**)

**Table S1. Aboriginal and Torres Strait Islander and non-Indigenous population (five State/Territory) across periods, and change from 2001 to 2016, by age group and sex**

|  |  | **Aboriginal and Torres Strait Islander population** | | | | |  | **Non-Indigenous population** | | | | |
| --- | --- | --- | --- | --- | --- | --- | --- | --- | --- | --- | --- | --- |
| **Persons** |  | **2001** | **2006** | **2011** | **2016** | **Δ 2001 to 2016** |  | **2001** | **2006** | **2011** | **2016** | **Δ 2001 to 2016** |
| 0 |  | 14 091 | 14 896 | 14 218 | 16 573 | 2482 |  | 167 364 | 176 929 | 194 962 | 208 102 | 40 738 |
| 1 - 4 |  | 55 248 | 56 106 | 58 912 | 66 119 | 10 871 |  | 678 700 | 682 962 | 781 976 | 823 092 | 144 392 |
| 5 - 14 |  | 119 002 | 133 826 | 139 816 | 159 549 | 40 547 |  | 1 806 862 | 1 815 764 | 1 865 635 | 1 989 030 | 182 168 |
| 15 - 24 |  | 83 340 | 99 622 | 117 679 | 136 704 | 53 364 |  | 1 788 138 | 1 917 467 | 2 055 167 | 2 089 030 | 300 892 |
| 25 - 34 |  | 75 482 | 74 476 | 80 800 | 100 589 | 25 107 |  | 1 940 998 | 1 947 971 | 2 175 429 | 2 417 843 | 476 845 |
| 35 - 44 |  | 59 829 | 67 980 | 72 653 | 77 972 | 18 143 |  | 2 041 431 | 2 078 109 | 2 171 943 | 2 201 592 | 160 161 |
| 45 - 54 |  | 36 745 | 46 853 | 55 757 | 70 995 | 34 250 |  | 1 845 061 | 1 968 660 | 2 114 546 | 2 162 611 | 317 550 |
| 55 - 64 |  | 17 985 | 24 215 | 32 299 | 46 258 | 28 273 |  | 1 277 058 | 1 574 227 | 1 798 766 | 1 930 270 | 653 212 |
| 65 - 74 |  | 9568 | 10 773 | 13 744 | 21 178 | 11 610 |  | 916 522 | 979 945 | 1 185 311 | 1 459 600 | 543 078 |
| 75 and over |  | 3846 | 5055 | 6080 | 8347 | 4501 |  | 776 026 | 887 013 | 983 929 | 1 107 055 | 331 029 |
| **Males** |  |  |  |  |  |  |  |  |  |  |  |  |
| 0 |  | 7194 | 7561 | 7180 | 8560 | 1366 |  | 85 975 | 90 726 | 99 837 | 106 824 | 20 849 |
| 1 - 4 |  | 28 268 | 28 644 | 30 095 | 34 086 | 5818 |  | 348 090 | 351 149 | 401 507 | 421 883 | 73 793 |
| 5 - 14 |  | 61 162 | 68 729 | 71 273 | 81 431 | 20 269 |  | 927 268 | 931 394 | 957 360 | 1 021 019 | 93 751 |
| 15 - 24 |  | 41 575 | 50 462 | 60 316 | 70 086 | 28 511 |  | 912 518 | 980 256 | 1 052 541 | 1 068 215 | 155 697 |
| 25 - 34 |  | 36 608 | 36 453 | 40 051 | 50 744 | 14 136 |  | 968 519 | 977 734 | 1 098 798 | 1 207 313 | 238 794 |
| 35 - 44 |  | 29 281 | 32 643 | 34 861 | 38 037 | 8756 |  | 1 017 213 | 1 035 893 | 1 082 121 | 1 097 890 | 80 677 |
| 45 - 54 |  | 18 126 | 22 958 | 26 836 | 33 468 | 15 342 |  | 925 090 | 978 630 | 1 050 976 | 1 066 111 | 141 021 |
| 55 - 64 |  | 8644 | 11 608 | 15 473 | 21 877 | 13 233 |  | 649 578 | 792 309 | 898 262 | 948 015 | 298 437 |
| 65 - 74 |  | 4318 | 4844 | 6275 | 9884 | 5566 |  | 445 499 | 480 922 | 588 771 | 722 800 | 277 301 |
| 75 and over |  | 1699 | 2030 | 2393 | 3338 | 1639 |  | 305 791 | 363 449 | 412 520 | 481 920 | 176 129 |
| **Females** |  |  |  |  |  |  |  |  |  |  |  |  |
| 0 |  | 6897 | 7335 | 7038 | 8013 | 1116 |  | 81 389 | 86 203 | 95 125 | 101 278 | 19 889 |
| 1 - 4 |  | 26 980 | 27 462 | 28 817 | 32 033 | 5053 |  | 330 610 | 331 813 | 380 469 | 401 209 | 70 599 |
| 5 - 14 |  | 57 840 | 65 097 | 68 543 | 78 118 | 20 278 |  | 879 594 | 884 370 | 908 275 | 968 011 | 88 417 |
| 15 - 24 |  | 41 765 | 49 160 | 57 363 | 66 618 | 24 853 |  | 875 620 | 937 211 | 1 002 626 | 1 020 815 | 145 195 |
| 25 - 34 |  | 38 874 | 38 023 | 40 749 | 49 845 | 10 971 |  | 972 479 | 970 237 | 1 076 631 | 1 210 530 | 238 051 |
| 35 - 44 |  | 30 548 | 35 337 | 37 792 | 39 935 | 9387 |  | 1 024 218 | 1 042 216 | 1 089 822 | 1 103 702 | 79 484 |
| 45 - 54 |  | 18 619 | 23 895 | 28 921 | 37 527 | 18 908 |  | 919 971 | 990 030 | 1 063 570 | 1 096 500 | 176 529 |
| 55 - 64 |  | 9341 | 12 607 | 16 826 | 24 381 | 15 040 |  | 627 480 | 781 918 | 900 504 | 982 255 | 354 775 |
| 65 - 74 |  | 5250 | 5929 | 7469 | 11 294 | 6044 |  | 471 023 | 499 023 | 596 540 | 736 800 | 265 777 |
| 75 and over |  | 2147 | 3025 | 3687 | 5009 | 2862 |  | 470 235 | 523 564 | 571 409 | 625 135 | 154 900 |

**Table S2. Aboriginal and Torres Strait Islander and non-Indigenous deaths across periods, and change from 2001 to 2016, by age group and sex**

|  |  | **Aboriginal and Torres Strait Islander population** | | | | |  | **Non-Indigenous population** | | | | |
| --- | --- | --- | --- | --- | --- | --- | --- | --- | --- | --- | --- | --- |
| **Persons** |  | **2001** | **2006** | **2011** | **2016** | **Δ 2001 to 2016** |  | **2001** | **2006** | **2011** | **2016** | **Δ 2001 to 2016** |
| 0 |  | 116 | 112 | 105 | 98 | -18 |  | 823 | 790 | 725 | 595 | -228 |
| 1 - 4 |  | 27 | 19 | 19 | 15 | -12 |  | 169 | 156 | 130 | 131 | -38 |
| 5 - 14 |  | 20 | 26 | 33 | 30 | 10 |  | 229 | 163 | 175 | 153 | -76 |
| 15 - 24 |  | 102 | 113 | 135 | 105 | 3 |  | 985 | 884 | 735 | 700 | -285 |
| 25 - 34 |  | 174 | 167 | 151 | 198 | 24 |  | 1391 | 1153 | 1196 | 1261 | -130 |
| 35 - 44 |  | 273 | 287 | 284 | 279 | 6 |  | 2314 | 2073 | 2080 | 2161 | -153 |
| 45 - 54 |  | 288 | 341 | 380 | 457 | 169 |  | 4341 | 4426 | 4667 | 4541 | 200 |
| 55 - 64 |  | 335 | 350 | 439 | 584 | 249 |  | 7917 | 8394 | 9237 | 9389 | 1472 |
| 65 - 74 |  | 321 | 344 | 378 | 537 | 216 |  | 15 985 | 14 107 | 15 317 | 17 192 | 1207 |
| 75 and over |  | 289 | 374 | 463 | 614 | 325 |  | 52 602 | 59 748 | 66 998 | 72 802 | 20 200 |
| **Males** |  |  |  |  |  |  |  |  |  |  |  |  |
| 0 |  | 70 | 63 | 56 | 48 | -22 |  | 466 | 462 | 395 | 341 | -125 |
| 1 - 4 |  | 15 | 12 | 11 | 9 | -6 |  | 100 | 80 | 62 | 77 | -23 |
| 5 - 14 |  | 12 | 16 | 17 | 17 | 5 |  | 148 | 99 | 96 | 89 | -59 |
| 15 - 24 |  | 70 | 73 | 91 | 72 | 2 |  | 744 | 639 | 496 | 503 | -241 |
| 25 - 34 |  | 122 | 114 | 94 | 123 | 1 |  | 1035 | 835 | 861 | 835 | -200 |
| 35 - 44 |  | 168 | 189 | 163 | 171 | 3 |  | 1459 | 1350 | 1317 | 1384 | -75 |
| 45 - 54 |  | 169 | 196 | 212 | 269 | 100 |  | 2720 | 2716 | 2884 | 2820 | 100 |
| 55 - 64 |  | 201 | 199 | 254 | 330 | 129 |  | 5138 | 5325 | 5908 | 5899 | 761 |
| 65 - 74 |  | 192 | 187 | 197 | 276 | 84 |  | 9978 | 8817 | 9449 | 10 732 | 754 |
| 75 and over |  | 134 | 164 | 217 | 254 | 120 |  | 23 669 | 27 080 | 30 636 | 33 864 | 10195 |
| **Females** |  |  |  |  |  |  |  |  |  |  |  |  |
| 0 |  | 46 | 49 | 49 | 50 | 4 |  | 357 | 328 | 330 | 254 | -103 |
| 1 - 4 |  | 12 | 7 | 8 | 6 | -6 |  | 69 | 76 | 68 | 54 | -15 |
| 5 - 14 |  | 8 | 10 | 16 | 13 | 5 |  | 81 | 64 | 79 | 64 | -17 |
| 15 - 24 |  | 32 | 40 | 44 | 33 | 1 |  | 241 | 245 | 239 | 197 | -44 |
| 25 - 34 |  | 52 | 53 | 57 | 75 | 23 |  | 356 | 318 | 335 | 426 | 70 |
| 35 - 44 |  | 105 | 98 | 121 | 108 | 3 |  | 855 | 723 | 763 | 777 | -78 |
| 45 - 54 |  | 119 | 145 | 168 | 188 | 69 |  | 1621 | 1710 | 1783 | 1721 | 100 |
| 55 - 64 |  | 134 | 151 | 185 | 254 | 120 |  | 2779 | 3069 | 3329 | 3490 | 711 |
| 65 - 74 |  | 129 | 157 | 181 | 261 | 132 |  | 6007 | 5290 | 5868 | 6460 | 453 |
| 75 and over |  | 155 | 210 | 246 | 360 | 205 |  | 28 933 | 32 668 | 36 362 | 38 938 | 10 005 |

Mortality rate data are restricted to the five of eight Australian States/Territories where the quality of Indigenous identification is deemed adequate (New South Wales, Queensland, South Australia, Western Australia, Northern Territory).

**Table S3. Aboriginal and Torres Strait Islander and non-Indigenous) age-specific and age-standardised mortality rates by age group, 2006 and 2011**

| **Persons** | **Aboriginal and Torres Strait Islander mortality rate (95% CI)** | |  | **Non-Indigenous mortality rate (95% CI)** | |
| --- | --- | --- | --- | --- | --- |
|  | **2006** | **2011** |  | **2006** | **2011** |
| **Age-specific mortality rates** |  |  |  |  |  |
| 0 | 752 (619, 905) | 739 (604, 894) |  | 447 (416, 479) | 372 (345, 400) |
| 1 - 4 | 34 (20, 53) | 32 (19, 50) |  | 23 (19, 27) | 17 (14, 20) |
| 5 - 14 | 19 (13,28) | 24 (16, 33) |  | 9 (8, 10) | 9 (8, 11) |
| 15 - 24 | 113 (93, 136) | 115 (96, 136) |  | 46 (43, 49) | 36 (33, 38) |
| 25 - 34 | 224 (192, 261) | 187 (158, 219) |  | 59 (56, 63) | 55 (52, 58) |
| 35 - 44 | 422 (375, 474) | 391 (347, 439) |  | 100 (96, 104) | 96 (92, 100) |
| 45 - 54 | 728 (632, 809) | 682 (615, 754) |  | 225 (218, 232) | 221 (214, 227) |
| 55 - 64 | 1445 (1298, 1605) | 1359 (1235, 1492) |  | 533 (522, 545) | 514 (503, 524) |
| 65 - 74 | 3193 (2865, 3549) | 2750 (2480, 3042) |  | 1440 (1416, 1464) | 1292 (1272, 1313) |
| 75 and over | 7399 (6668, 8188) | 7615 (6937, 8341) |  | 6736 (6682, 6790) | 6809 (6758, 6861) |
| **Mortality rates for all ages combined** |  |  |  |  |  |
| All ages (crude) | 400 (383, 417) | 403 (387, 420) |  | 655 (651, 659) | 661 (657, 665) |
| Age-standardised (Indigenous Standard) | 482 (461, 503) | 453 (435, 471) |  | 227 (225, 229) | 216 (215, 218) |
| Age-standardised (Australian Standard) | 1006 (954, 1060) | 964 (917, 1012) |  | 604 (600, 608) | 592 (589, 596) |

Mortality rate data are restricted to the five of eight Australian States/Territories where the quality of Indigenous identification is deemed adequate (New South Wales, Queensland, South Australia, Western Australia, Northern Territory).

**Table S4. Male Aboriginal and Torres Strait Islander and non-Indigenous age-specific and age-standardised mortality rates by age group, 2001 and 2016**

| **Males** | **Aboriginal and Torres Strait Islander mortality rate (95%CI)** | |  | **Non-Indigenous mortality rate (95%CI)** | |
| --- | --- | --- | --- | --- | --- |
|  |  |  |  |  |  |
|  | **2001** | **2016** |  | **2001** | **2016** |
| **Age-specific mortality rates** |  |  |  |  |  |
| 0 | 973 (759, 1229) | 561 (413, 743) |  | 542 (494, 594) | 319 (286, 355) |
| 1 - 4 | 53 (30, 88) | 26 (12, 50) |  | 29 (23, 35) | 18 (14, 23) |
| 5 - 14 | 20 (10, 34) | 21 (12, 33) |  | 16 (13, 19) | 9 (7, 11) |
| 15 - 24 | 168 (131, 213) | 103 (80, 129) |  | 82 (76, 88) | 47 (43, 51) |
| 25 - 34 | 333 (277, 398) | 242 (201, 289) |  | 107 (100, 114) | 69 (65, 74) |
| 35 - 44 | 574 (490, 667) | 450 (385, 522) |  | 143 (136, 151) | 126 (120, 133) |
| 45 - 54 | 932 (797, 1084) | 804 (711, 906) |  | 294 (283, 305) | 265 (255, 274) |
| 55 - 64 | 2325 (2015, 2670) | 1508 (1350, 1680) |  | 791 (769, 813) | 622 (606, 638) |
| 65 - 74 | 4447 (3840, 5122) | 2792 (2473, 3142) |  | 2240 (2196, 2284) | 1485 (1457, 1513) |
| 75 and over | 7887 (6608, 9341) | 7609 (6702, 8605) |  | 7740 (7642, 7840) | 7027 (6952, 7102) |
| **Age-standardised mortality rates** |  |  |  |  |  |
| All ages (crude) | 487 (459, 516) | 446 (425, 469) |  | 690 (684, 697) | 694 (689, 700) |
| Age-standardised (Indigenous Standard) | 655 (616, 695) | 483 (459, 507) |  | 310 (307, 314) | 243 (240, 245) |
| Age-standardised (Australian Standard) | 1281 (1186, 1381) | 1010 (948, 1075) |  | 771 (764, 779) | 642 (636, 647) |

Mortality rate data are restricted to the five of eight Australian States/Territories where the quality of Indigenous identification is deemed adequate (New South Wales, Queensland, South Australia, Western Australia, Northern Territory).

**Table S5. Female Aboriginal and Torres Strait Islander and non-Indigenous age-specific and age-standardised mortality rates by age group, 2001 and 2016**

| **Females** | **Aboriginal and Torres Strait Islander mortality rate (95%CI)** | |  | **Non-Indigenous mortality rate (95%CI)** | |
| --- | --- | --- | --- | --- | --- |
|  |  |  |  |  |  |
|  | **2001** | **2016** |  | **2001** | **2016** |
| **Age-specific mortality rates** |  |  |  |  |  |
| 0 | 667 (448, 890) | 624 (463, 823) |  | 439 (394, 487) | 251 (221, 284) |
| 1 - 4 | 44 (23, 78) | 19 (7, 41) |  | 21 (16, 26) | 13 (10, 18) |
| 5 - 14 | 14 (6, 27) | 17 (9, 28) |  | 9 (7, 11) | 7 (5, 8) |
| 15 - 24 | 77 (52, 108) | 50 (34, 70) |  | 28 (24, 31) | 19 (17, 22) |
| 25 - 34 | 134 (100, 175) | 150 (118, 189) |  | 37 (33, 41) | 35 (32, 39) |
| 35 - 44 | 344 (281, 416) | 270 (222, 327) |  | 83 (78, 89) | 70 (66, 76) |
| 45 - 54 | 639 (529, 765) | 501 (432, 578) |  | 176 (168, 185) | 157 (150, 165) |
| 55 - 64 | 1435 (1202, 1699) | 1042 (918, 1178) |  | 443 (427, 460) | 355 (344, 367) |
| 65 - 74 | 2457 (2051, 2920) | 2311 (2039, 2609) |  | 1275 (1243, 1308) | 877 (856, 898) |
| 75 and over | 7219 (6128, 8450) | 7187 (6464, 7969) |  | 6153 (6082, 6224) | 6229 (6167, 6291) |
| **Age-standardised mortality rates** |  |  |  |  |  |
| All ages (crude) | 332 (310, 356) | 382 (362, 403) |  | 621 (615, 627) | 635 (630, 641) |
| Age-standardised (Indigenous Standard) | 417 (387, 448) | 358 (339, 378) |  | 195 (192, 197) | 167 (165, 169) |
| Age-standardised (Australian Standard) | 900 (825, 981) | 819 (769, 870) |  | 536 (531, 541) | 496 (492, 500) |

Mortality rate data are restricted to the five of eight Australian States/Territories where the quality of Indigenous identification is deemed adequate (New South Wales, Queensland, South Australia, Western Australia, Northern Territory).

references

1. Ulm K. A simple method to calculate the confidence interval of a standardised mortality ratio. *American journal of epidemiology* 1990;**131**: 373-5.

2. Australian Bureau of Statistics. 2071.0 - Census of Population and Housing: Reflecting Australia - Stories from the Census, 2016. <http://www.abs.gov.au/ausstats/abs@.nsf/mf/2071.0> (Accessed 27 Feb 2020). Canberra: ABS; 2017.

3. Sahai H, Khurshid A. Statistics in epidemiology: methods, techniques, and applications. Boca Raton: CRC Press; 1996.

4. Kumar M. Directly Standardized Rates. Available from: <https://cran.r-project.org/web/packages/dsr/vignettes/dsr.html> [Accessed 19 Aug 2020]. 2019.

5. MedCalc Software Ltd. MEDCALC: easy-to-use statistical software. Available from: <https://www.medcalc.org/manual/comparison_of_rates.php> [Accessed 19 Aug 2020]. 2020.
